# Comparison of duplex PCR and phenotypic analysis in differentiating *Candida dubliniensis* from *Candida albicans* from oral samples

**DOI:** 10.1186/s13568-017-0435-9

**Published:** 2017-06-27

**Authors:** Asanga Sampath, Manjula Weerasekera, Ayomi Dilhari, Chinthika Gunasekara, Uditha Bulugahapitiya, Neluka Fernando, Lakshman Samaranayake

**Affiliations:** 1 Department of Microbiology, Faculty of Medical Sciences, University of Sri Jayewardenepura, Gangodawila, Nugegoda, Sri Lanka; 2 Diabetes and Endocrinology Unit, Colombo South Teaching Hospital, Kalubowila, Dehiwala, Sri Lanka; 3 Health Science Center, Kuwait University, Kuwait City, Kuwait

**Keywords:** *Candida albicans*, *Candida dubliniensis*, Duplex PCR, Diabetes, Oral rinse

## Abstract

*Candida dubliniensis* shares a wide range of phenotypic characteristics with *Candida albicans* including a common trait called germ tube positivity. Hence, laboratory differentiation of these two species is cumbersome. Duplex PCR analyses for *C. albicans* and *C. dubliniensis* was performed directly on DNA extracted from a total of 122 germ tube positive isolates derived from 100 concentrated oral rinse samples from a random cohort of diabetics attending a clinic in Sri Lanka. These results were confirmed by DNA sequencing of internal transcribed spacer (ITS) region of rDNA of the yeasts. Performance efficacy of duplex PCR was then compared with phenotypic identification using a standard battery of phenotypic tests. Of the 122 germ tube positive isolates three were identified by duplex PCR as *C. dubliniensis* and the remainder as *C. albicans.* On the contrary, when the standard phenotypic tests, sugar assimilation and chlamydospore formation, were used to differentiate the two species 13 germ tube positive isolates were erroneously identified as *C. dubliniensis*. Duplex PCR was found to be rapid, sensitive and more specific than phenotypic identification methods in discriminating *C. dubliniensis* from *C. albicans.* This is also the first report on the oral carriage of *C. dubliniensis* in a Sri Lankan population.

## Introduction

*Candida dubliniensis* is an emerging medically relevant pathogenic yeast (Sullivan et al. 1995) associated with oral, vaginal, and systemic infections particularly in patients with human immunodeficiency virus infection and diabetes mellitus (Krcmery and Barnes 2002; Sullivan et al. 1995). *C. dubliniensis* and *C. albicans* are known to share many morphological and physiological characteristics such as germ tube positivity, production of chlamydospores and similar biochemical profiles leading to common misidentification of these two species (Sullivan and Coleman 1998; Sullivan et al. 1995). Also, variations in growth conditions including incubation temperature, repeated sub-culture, and storage may impede their accurate identification (Pasligh et al. 2008). Furthermore, both these species are now known to be increasingly resistant to commonly used azole antifungal agents. Given their importance in common oral and systemic infections, there is a critical need to accurately and rapidly identify these pathogens for better patient management.

Several phenotypic and genotypic tests have been developed, validated and applied to differentiate *C. albicans* from *C. dubliniensis.* Commercially available yeast identification systems (e.g. Vitek 2 ID-YST, API 20C and ID32C) based on utilization of various compounds are the most popular current methods used in clinical laboratories for this purpose, although they are relatively expensive and requires up to 2–3 days incubation to obtain results (Pincus et al. 1999). On the other hand, polymerase chain reaction PCR (Ellepola et al. 2003) and duplex PCR (Ahmad et al. 2012) have been recently used as genotypic tests for rapid identification and differentiation of the two species.

Here we report a comparison of well described phenotypic methods with the duplex PCR assay (Ahmad et al. 2012) using primers derived from unique ribosomal deoxyribonucleic acid (rDNA) sequences for rapid detection and differentiation of *C. albicans* and *C. dubliniensis*. The was assays were performed using yeast isolates derived from oral rinse samples from a cohort of diabetic patients attending a clinical facility in Colombo, Sri Lanka.

## Materials and methods

### Patients and yeast isolates

Concentrated oral rinse samples were randomly collected from 250 patients with type 2 diabetes mellitus, attending the Diabetes and Endocrinology Unit at a Tertiary Care Hospital in Sri Lanka, as per the method of Samaranayake et al. (1986). In brief, the patients were given 10 ml of sterile phosphate-buffered saline and advised to rinse their mouths for 60 s and expectorate into the provided container. Each specimen was immediately taken to the laboratory, vortex mixed and centrifuged at (6000 rpm) 3300×*g* for 10 min. The pellet obtained from the rinse specimen was re-suspended in 1 ml of sterile phosphate buffered saline. Hundred microliter of the re-suspended specimens was cultured on Sabouraud Dextrose Agar and incubated at 37 °C for 72 h and the resultant growth evaluated for yeast growth.

Subsequently, one to two colony phenotypes (colony forming units, CFUs) that resemble different yeast species were randomly selected from each sample. These CFUs were subcultured for 18 h to obtain a pure growth and harvested, and phenotypic and genotypic analysis performed to differentiate the two species, as described below.

For standardization purposes two reference isolates: *C. albicans* American Type Culture Collection (ATCC) 10231, *C. dubliniensis* ATCC MYA 580 and *C. dubliniensis* ATCC MYA 577 obtained from the Department of Oral Biology, University of Hong Kong, China, were used.

### Phenotypic analysis

Of the 250 patient samples collected, 204 (81.6%) yielded a yeast growth on culture. The culture plates were then examined by a single examiner and up to two different phenotypes form each sample were selected for the germ tube test (Isibor et al. 2005). The final analysis was then performed as follows: first, the rinse pellets derived from randomly selected 100 samples that yielded germ tube positive yeasts were subjected to duplex PCR. Then, 122 germ tube positive yeast isolates from the aforementioned (100) samples were subjected to both phenotypic analysis and genotypic analysis (i.e. duplex PCR) as described below. The phenotypic tests used for speciation of 122 isolates were chlamydospore production test (Kim et al. 2002), growth at 42 °C (Sullivan et al. 1995), and assimilation of xylose and trehalose (Pincus et al. 1999).

### DNA extraction, duplex PCR assay and sequence identification

DNA extraction was performed using the conventional bead beater method (Sambrook and Rusell 2006) with modifications. A loopful of isolated colonies or pellet of the concentrated oral rinse sample was suspended in 100 µl STES buffer [200 mM Tris–HCl (pH 7.6), 100 mM EDTA (Ethylenediaminetetraacetic acid), 0.1% SDS (sodium dodecyl sulfate)] and 40 µl of TE (Tris–EDTA) buffer [10 mM Tris–HCl (pH 8), 1 mM EDTA], 120 µl phenol: chloroform mixture (1:1 V/V) and 0.3 g sterile zirconium beads (0.1 mm diameter; Bio Spec-Products) were added. The samples were homogenized using a mini bead beater (model 3110BX; Bio Spec Products) at 480 rpm for 5 min. The upper aqueous phase (100 µl) was transferred to a sterile micro centrifuge tube, and DNA was precipitated in the presence of 220 µl cold ethanol (100%) and 10 µl of 3 M sodium acetate at −20 °C for 18 h. The solution was centrifuged at (13,000 rpm) 15,493×*g* for 12 min and the DNA pellet was air dried and dissolved in 30 µl TE buffer. Extracted DNA samples were stored at −20 °C until used.

Extracted DNA were subjected to the quantification using NANO drop 2000/200C spectrophotometer (Thermo Fisher Scientific, USA). Extracted DNA from oral rinse specimens and germ tube positive isolates were subjected to duplex PCR to differentiate *C. albicans* from *C. dubliniensis*.

Species-specific identity of *C. albicans* and *C. dubliniensis* strains were performed by duplex PCR using primers targeting sequences in *ITS*-*1* (internal transcribed spacer-1) and *ITS*-*2* regions of rDNA (Ahmad et al. 2012), respectively. Species-specificity of primer pairs for *C. albicans* and *C. dubliniensis* are shown in Table 1.

**Table 1:** The primer sequences used for amplification of *C. albicans* and *C. dubliniensis* for duplex PCR

Target gene	Target species	Primer	Primer sequence (5′–>3′)	Amplicon size (bp)
*ITS 2*	*C. albicans*	*CALF*	TGG TAA GGC GGG ATC GCT T	100
		*CALR*	GGT CAA AGT TTG AAG ATA TAC	
*ITS 1*	*C. dubliniensis*	*CDLF*	AAA CTT GTC ACG AGA TTA TTT TT	325
*ITS 2*		*CDLR*	AAA GTT TGA AGA ATA AAA TGG C	

Duplex PCR amplification was carried out in a final volume of 50 μl with 2 µl template DNA, 1× green Go Taq Flexi buffer (pH 8.5), 3 mM MgCl_2_, 0.2 µM of each primer (*CALF* + *CALR* + *CDUF* + *CDUR*) (Table 1), 0.2 mM Deoxy Nucleotide Triphosphate (dNTP) mix and 1.25 unit of Go Taq DNA polymerase (Promega, USA). PCR amplification was done using GeneAmp PCR systems 9700 (Applied Bio systems, USA). The PCR reaction was initiated at 95 °C for 5 min followed by 30 cycles of 95 °C for 1 min, 55 °C for 30 s and 72 °C for 1 min and a final elongation at 72 °C for 10 min with final hold at 4 °C. All PCR experiments included a negative (no template) control and a positive control. Resulting PCR products were separated by electrophoresis using 1× TAE (Tris base, acetic acid and EDTA) (40 mM Tris HCl (pH 8), 20 mM acetic acid, 1 mM EDTA) on a 3% (w/v) agarose gel, stained with ethidium bromide and viewed by UV (Ultra Violet) trans-illuminator (Vilber Lourmat, QUANTUM ST4).

The PCR products derived from isolates which were identified as *C. dubliniensis* by duplex PCR were purified using a pureLink™ Quick Gel Extraction and PCR Purification Combo Kit (Invitrogen, Thermo Fisher Scientific, and USA) and were subjected to sequencing using PCR primers in both directions. The DNA sequences obtained were aligned using BioEdit and subjected to National Center for Biotechnology Information (NCBI) Blast to identify the species.

## Results

### Clinical data

Of the 250 oral rinse samples collected, 204 (81.6%) yielded a yeast growth on culture. When the germ tube test was conducted 167 (81.8%) of the latter cohort were carrying germ tube positive yeasts, and the remainder germ tube negative yeasts. We then randomly selected 100 patient samples which were germ tube positive, and this cohort was carrying 122 germ tube positive yeast isolates that were selected for the final analysis (some patients carried two, germ tube positive phenotypes). The rinse pellets derived from the foregoing samples were also subjected to duplex PCR in parallel. It is also noteworthy, that out of the selected 100 culture positive patients majority 67 per cent (67/100) had yeast colony counts over 600 colony-forming units (CFU) per ml of the concentrated oral rinse sample.

### Duplex PCR of the concentrated oral rinse samples

When the extracted DNA from pellets of 100 concentrated rinse samples were directly subjected to duplex PCR three samples were positive for *C. dubliniensis* and the remainder were positive for *C. albicans* only. Interestingly, of the three *C. dubliniensis* positive patients one patient yielded both *C. albicans* and *C. dubliniensis* and two patients yielded only *C. dubliniensis*.

**Fig. 1:**
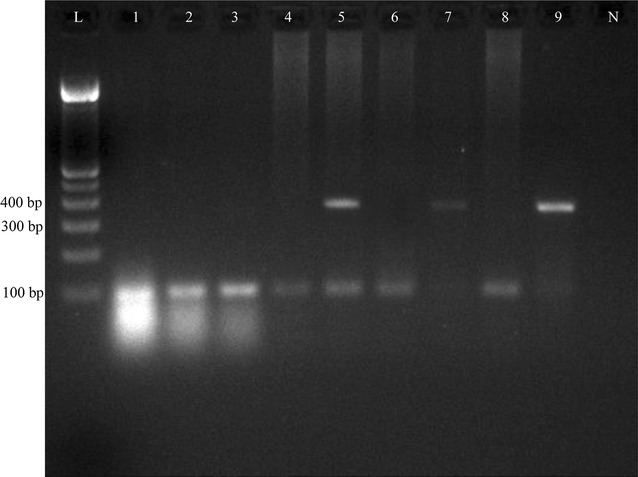
Duplex PCR results on the 2% Agarose gel. L, 100 bp ladder; N, negative control; *lane no 1–4*, *6* and *8*, *C. albicans*; *lanes 7* and *9*, *C. dubliniensis*; *lane 5*, positive for both *C. albicans* and *C. dubliniensis*

On duplex PCR, *C. dubliniensis* yielded a band around 325 bp positions while *C. albicans* gave a band at 100 bp position (Fig. 1).

### Duplex PCR of the germ tube positive isolates

The colonial yield from the 100 randomly selected patient samples were phenotypically evaluated for germ tube positivity a total of 122 germ tube positive strains were identified, and these were presumptively identified as either *C. albicans* or *C. dubliniensis*. When these were subjected to duplex PCR, three were identified as *C. dubliniensis* and the remainder as *C. albicans.*

Thus, the duplex PCR results from the pellet analysis of 100 patients or from analyzing the 122 germ tube positive yeasts derived from the same cohort were identical with three results that were positive.

### Phenotypic identification

Of the 122 germ tube positive isolates those were subjected to sugar assimilation tests using xylose and trehalose, to differentiate *C. albicans* from *C. dubliniensis*, xylose was assimilated by 108 isolates, trehalose by 114, and xylose and trehalose by 106 isolates. On this basis 16 isolates could be characterized as *C. dubliniensis*. However, when the identity of these 16 isolates were scrutinized by duplex PCR only three isolates were genotypically confirmed as *C. dubliniensis.*

On further analyzing the phenotype, of 122 isolates, 114 grew well at 42 °C and were identified as *C. albicans* and eight colonies which did not yield any growth at 42 °C and were presumptively identified as *C. dubliniensis.* However, when they were subjected to duplex PCR only one out of the eight isolates was definitively identified as *C. dubliniensis.* The test sensitivity and specificity of each phenotypic identification method was found to vary (Table 2).

**Table 2:** The sensitivity and the specificity of the phenotypic methods used for differentiating *C. albicans* and *C. dubliniensis* (using duplex PCR identification as the gold standard)

Cultivation method	*C. dubliniensis*—sensitivity (%)	*C. albicans*—specificity (%)	PPV (%)	NPV (%)
Chlamydospore formation	33.3	97.48	25	98.31
Growth at 42 °C	33.3	94.12	12.5	98.25
Assimilation of xylose	33.3	89.08	7.14	98.15
Assimilation of trehalose	33.3	94.12	12.5	98.25

*PPV* positive predictive value, *NPV* negative predictive value

### Comparison of duplex PCR and phenotypic identification

Comparing duplex PCR results, considered as the gold standard, with the phenotypic methods, the latter had poor sensitivity, of 33.3%, and relatively high specificity, and a poor positive predictive value and high negative predicative values (Table 2).

## Discussion

Oral infections with *Candida species* are on the rise. This is mainly due to the burgeoning immune compromised populations worldwide, such as those with human immunodeficiency virus (HIV) disease, and those on immune- and cytotoxic therapy. Additionally, the pre diabetics and diabetics are well known to have a high prevalence of oral *Candida* (Samaranayake 2011). Hence a rapid, reliable and an inexpensive method for identification of *Candida*, particularly *C. albicans,* the most virulent *Candida* species, from the less virulent surrogate, *C. dubliniensis* is clinically advantageous.

The duplex PCR assay described in this study enabled the accurate differentiation and identification of *C. dubliniensis* from *C. albicans* strains form oral rinses specimens. In total, the whole procedure could be completed in 4 h as opposed to up to 3 days required for the traditional, phenotypic tests. Further it requires minimal quantity of genomic DNA. Although this is not the first study to use duplex PCR to identify and distinguish *C. dubliniensis* and *C. albicans*, but it is the first to analyses oral samples directly through rinse pellet analysis, and samples from a Sri Lankan diabetic population using duplex PCR.

Ahmad et al. reported duplex PCR application as an accurate tool for identification and differentiation of *C. albicans* and *C. dubliniensis*, with a very high index of sensitivity and specificity (Ahmad et al. 2012). In agreement, with the flatter findings we too noted that duplex PCR amplification with *CALF* + *CALR* + *CDUF* + *CDUR* primers yielded species-specific single amplicons of ~100 and ~325 bp of *C. albicans* and *C. dubliniensis,* respectively, that provided a high degree of discrimination. Further, duplex PCR was found to be a rapid, sensitive and simple technique, which could be directly applied to oral rinse specimens as well as clinical isolates derived from these rinses. It may be possible to extend the application to the other clinical specimens such as blood, saliva and fecal specimens, especially in septicemic states with important clinical impact and further studies are warranted for this purpose.

In the current study, the results of duplex PCR assay were not fully in agreement with species-specific phenotypic identification of *C. dubliniensis* and *C. albicans.* For instance, the growth at 42 °C poorly differentiated the two species. Others too have previously reported that growth at a higher temperature as an unreliable criterion for this purpose (Pasligh et al. 2010). Similarly, the assimilation data for trehalose and xylose also were not reliable and confirmed the findings of Tintelnot et al. who concluded that the observed pattern for the assimilation of xylose is not discriminating enough to differentiate the two species (Tintelnot et al. 2000). Finally, the corn meal and tween 80 agar tests to evaluate the degree of chlamydospore formation was also unreliable as one-third of both species produced these appendages (Table 2) This is consistent with the observations of previous workers who noted that both *C. dubliniensis* and *C. albicans* produce chlamydospores (Sancak et al. 2005; Sullivan and Coleman 1998; Sullivan et al. 1995) and querying its validity as a differential phenotypic trait.

In clinical terms, we noted that 67% of our Sri Lankan diabetic population yielded oral *Candida* concentrations greater than 600 CFU per ml as evaluated by concentrated oral rinse culture indicating a rather heavy carriage of oral yeasts, and the consequent probability of overt yeast infection. Candida cell counts of >600 CFU per ml in oral rinse samples is considered as indicative of oral *Candida* infection according to previous workers (Samaranayake and MacFarlane 1990).

Others have reported oral carriage of *Candida* in patients with type 2 diabetes mellitus ranging from 13 to 64% (Fernandez et al. 2013). Such wide variation in oral Candidal carriage is a reflection of many confounding factors including the diabetes status and more crucially, the sampling method. It is well known that oral rinses samples yield a higher CFU count than the swab sampling method. The latter method excludes various oral niches preferred by *Candida* such as the posterior vestibular sulci (Samaranayake and MacFarlane 1990).

One interesting observation of the study was a single patient with dual species oral carriage of both *C. albicans* and *C. dubliniensis.* Revelation of such dual species carriage of phenotypically similar yeast isolates would be extremely difficult, if not time consuming and labor intensive, by conventional cultural techniques. The duplex PCR would therefore be useful to uncover the little described phenomenon of the multi species oral yeast carriage. For instance, Samaranayake et al. (1987) in a similar study revealed, up to 15% of oral rinse specimens in a British dental hospital yielded more than one yeast species, and hence duplex as well as multiplex PCR technology should be used by future workers to shed further light on this phenomenon (Samaranayake et al. 1987).

Finally, to our knowledge, this is first report of *C. dubliniensis* isolation and oral carriage in a Sri Lankan cohort. The 3% prevalence of carriage reported here is similar to that of 2% oral and gastro intestinal prevalence reported by Odds et al. (1998) in 2589 yeasts in a stock collection from Europe, implying that *C. dubliniensis* is an opportunist pathogen of low consequence with similar worldwide prevalence profiles (Odds et al. 1998). Further clinical epidemiological data, particularly from the Asian region, are needed to confirm our preliminary findings.

## Abbreviations

PCR: polymerase chain reaction
DNA: deoxyribonucleic acid
ITS: internal transcribed spacer
rDNA: ribosomal deoxyribonucleic acid
ATCC: American Type Culture Collection
HCl: hydrochloric acid
EDTA: ethylenediaminetetraacetic acid
MgCl_2_: magnesium chloride
SDS: sodium dodecyl sulfate
TE: tris-EDTA buffer solution
dNTP: deoxy nucleoside tri-phosphate
UV: ultra violet
TAE: tris base, acetic acid and EDTA
NCBI: National Center for Biotechnology Information
CFU: colony-forming units
HIV: human immunodeficiency virus
